# Prognosis of Guillain–Barré Syndrome Linked to COVID-19 Vaccination

**DOI:** 10.3390/brainsci12060711

**Published:** 2022-05-30

**Authors:** Shaun Kai Kiat Chua, Qian Ying Soh, Seyed Ehsan Saffari, Eng-King Tan

**Affiliations:** 1 Lee Kong Chian School of Medicine, Nanyang Technological University, 11 Mandalay Rd, Singapore 30832, Singapore; schua041@e.ntu.edu.sg (S.K.K.C.); qsoh002@e.ntu.edu.sg (Q.Y.S.); 2 National Neuroscience Institute, Duke NUS Medical School, Outram Rd, Singapore 169608, Singapore; ehsan.saffari@duke-nus.edu.sg

**Keywords:** Guillain–Barré syndrome, COVID-19 vaccination, SARS-CoV-2 vaccination, prognosis

## Abstract

There have been increasing reports of Guillain–Barré syndrome (GBS), a rare but debilitating neurological disease, occurring post-COVID-19 vaccination. However, the outcomes and relationships between patient demographics and clinical outcomes of post-COVID-19 vaccination GBS remain unclear. To bridge this gap, our study investigates the outcomes and clinical factors associated with poorer GBS outcomes following COVID-19 vaccination. We conducted a review and pooled analysis of detailed data extracted from 57 published cases with the relevant search strategies and criteria. The groups compared included male versus female patients, 1st dose versus 2nd dose and early onset versus late onset of GBS. Multivariate regression analysis was performed to compare the vaccine type, clinical severity and post-treatment outcomes between these groups of patients. Our results highlight for the first time that females were significantly more likely to have severe clinical presentation and poorer outcomes compared to males. Additionally, viral vector vaccines were the predominant vaccine type administered in early-onset post-COVID-19-vaccination GBS and GBS occurring after the 1st vaccination dose. It was also shown that reported cases of post-vaccination GBS generally displayed a positive response to conventional treatment and had favourable post-treatment outcomes. Through this study, we have established important links and provided assuring evidence for treatment response and post-treatment outcomes of GBS occurring post-COVID-19 vaccination. While the COVID-19 vaccination brings about much greater benefits than risks, our findings provide further impetus for greater vigilance in certain patient groups and more studies to explore the mechanisms behind these links.

## 1. Introduction

Guillain–Barré syndrome (GBS) is a rare but debilitating inflammatory neurological disease of the peripheral neurological system, affecting around 0.4 to 1.7 persons per 100,000 population annually [1]. GBS has a heterogeneous clinical presentation characterized by features of symmetrical ascending limb weakness, sensory loss, areflexia and cranial nerve deficits, progressing to respiratory failure in severe cases [2]. Diagnosis of this disease requires a combination of clinical findings supported by nerve conduction studies (NCS) and cerebrospinal fluid (CSF) examination [3]. While the aetiology of GBS is unclear, its pathogenesis has been largely attributed to post-infectious and post-vaccination mechanism causes [2].

Since the global initiation of the Coronavirus Disease 2019 (COVID-19) vaccination programme, there have been increasing reports of GBS following the COVID-19 vaccination, alluding to possible associations between the two conditions [4]. Current studies have shown a higher risk of post-COVID-19 vaccination GBS in certain vaccine types, such as the Ad.26.COV2.S (Janssen) vaccine [5], while others have highlighted a higher incidence of other neurological adverse effects such as Myasthenia Gravis in the female gender and after the first vaccine dose [6]. In GBS cases with non-specific etiologies, patient demographic factors such as the female gender have been associated with poorer outcomes [7]. However, there remains a research gap with regards to the relationship between patient demographics and the clinical outcomes of GBS occurring specifically after COVID-19 vaccination. 

To bridge the gaps in knowledge, we investigated the outcomes and clinical factors associated with poorer GBS outcomes following COVID-19 vaccination through a pooled analysis of detailed data from published case reports.

## 2. Methods 

The search strategy was made in accordance with the Preferred Reporting Items for Systematic Reviews and Meta-Analyses (PRISMA) [8].

### 2.1. Search Strategy

An electronic database search on PubMed, EMBASE and Web of Science was performed by two independent authors (S.K.K.C and S.Q.Y) to identify published reports of post-COVID-19 vaccination GBS in English from 1 January 2021 to 1 November 2021. The search terms “Guillain–Barré syndrome”, “Miller Fisher syndrome”, “facial diplegia”, “cranial polyneuritis”, “acute inflammatory demyelinating polyneuropathy”, “acute motor and sensory axonal neuropathy”, “acute motor axonal neuropathy”, “acute sensory ataxia”, “Bickerstaff encephalitis”, “SARS-CoV-2 vaccine”, “SARS-CoV-2 vaccination”, “COVID-19 vaccine” and “COVID-19 vaccination” were used with appropriate Boolean operators for the identification of potential studies. The details of the search strategy are presented in Annex 1.

### 2.2. Eligibility Criteria

All published case reports and case series reporting cases of GBS post-COVID-19 vaccination, with symptom onset within 21 days of COVID-19 vaccination and a confirmatory diagnosis from cerebrospinal fluid (CSF) analysis or nerve conduction study (NCS) were included. Patients with a preceding history of GBS and articles not written in English or where access to the full text was unavailable were excluded.

The articles were independently assessed by two authors (S.K.K.C and S.Q.Y) to determine the eligibility to be included in the final review. Any disagreements were discussed and resolved by consensus among the authors. 

### 2.3. Data Extraction

Data regarding study details (study design, study location), patient demographics (age, gender, ethnicity, comorbidities, past medical history), vaccine information (type, brand and dose number of COVID-19 vaccine administered), duration of symptom onset, presenting symptoms, investigations, management and treatment outcomes were extracted. All reported outcomes were considered regardless of the timing from symptom onset or treatment. Where clinical symptoms were described at different time points, information representing the full disease was reported. Based on the reported symptoms, the cases were classified into different GBS clinical subtypes, including classic GBS, paraparetic GBS, Miller Fisher Syndrome (MFS), facial diplegia and cervicobrachial weakness [9].

### 2.4. Statistical Analysis

Multivariate regression analysis was performed to compare the vaccine type, clinical severity (defined by the presence of respiratory failure, use of ventilatory support, intensive care unit (ICU) admission, or death), and post-treatment outcomes (defined by GBS disability score [10]) between different groups of patients. The groups of patients compared were male versus female, 1st dose versus 2nd dose, and early onset (≤7 days) versus late-onset (≥8 days). Statistical significance was set at *p*-value <0.05, with adjusted odds ratios (OR) and 95% confidence intervals (CI) reported.

## 3. Results

### 3.1. Included Studies

**Figure 1:**
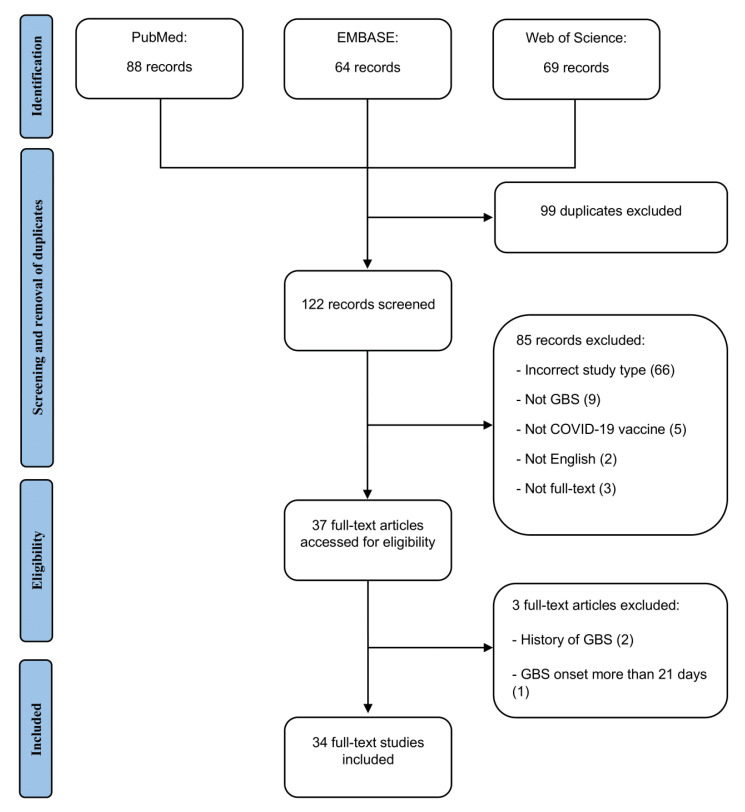
PRISMA flow diagram of search and selection process. GBS: Guillain–Barré Syndrome; COVID-19: coronavirus disease 2019.

Thirty-four articles, comprising 57 patients, were included in this study [4,11,12,13,14,15,16,17,18,19,20,21,22,23,24,25,26,27,28,29,30,31,32,33,34,35,36,37,38,39,40,41,42,43]. The search and selection process are detailed in Figure 1. Among the included patients, 16 (28%) were from Asian countries, including South Korea, Qatar, India and Taiwan, 4 (7%) were from Australia, 10 (18%) were from the United States (US), 19 (33%) were from European countries including the United Kingdom (UK), Turkey, Italy, Spain and Malta and 7 (12%) were from South America including Mexico and Brazil (Supplementary Table S1). Information regarding study details, patient demographics, vaccine information, duration of symptom onset, presenting symptoms, investigations, management and treatment outcomes are detailed in Supplementary Tables S1–S3.

### 3.2. Patient Demographics and Outcomes

The mean age of 57 patients was 59.5 years, with 31 (54%) being males. Thirty-nine (68%) received viral vector vaccines, and 46 (81%) occurred after administration of the first dose. The mean duration of GBS symptom onset post-COVID-19 vaccination was 10.7 ± 5.2 days. In terms of the clinical subtype of patients, 35 (61%) patients were of the classic GBS subtype and 9 (16%) patients were of the paraparetic subtype (Table 1). Twelve (21%) patients presented with the facial diplegia subtype, while 1 (2%) patient presented with cervicobrachial weakness (Table 1). The severe presentation was described in 17 (30%) patients, with 16 (28%) requiring ICU admission, 14 (25%) experiencing respiratory failure, 13 (23%) requiring ventilatory support, and 1 (2%) patient death reported.

Forty-nine (86%) patients were treated with intravenous immune globulin (IVIg), while 1 (2%) and 2 (4%) patients were, respectively, treated with plasmapheresis and combination therapy of plasmapheresis and IVIg. One (2%) patient each received gabapentin and oral prednisolone treatment, respectively, and 3 (5%) patients did not receive any treatment. After initiation of treatment, 34 (60%) reported definite improvement, 16 (28%) reported minimal improvement, 4 (7%) reported no improvement and 2 (4%) reported complete recovery. Post-treatment outcomes were reported in 51 (90%) patients, and 38 (67%) patients had a GBS disability scale score of 3 or less, indicating an ability to at least ambulate with assistance, while 17 (33%) patients had poorer outcomes with a GBS disability scale score of 4 and above indicating they were either bedbound, requiring ventilatory support, or dead (Table 1). Details of patient demographics, vaccine information, duration of symptom onset, presenting symptoms, investigations, management and treatment outcomes are summarized in Table 1.

### 3.3. Multivariate Analysis 

Our main finding from the multivariate analysis revealed that females presented with significantly greater clinical severity (OR = 0.26; 95% CI = [0.07, 0.91]; *p* value = 0.035) and poorer post-treatment outcomes (β = −1.41; 95% CI = [−2.27, −0.55]; *p* value = 0.0020). Additionally, viral vector vaccines were found to be the predominant vaccine type administered in early-onset post-COVID-19 vaccination GBS (OR = 11.1; 95% CI = [2.35, 52.1]; *p* value = 0.0020), and the primary vaccine type in GBS occurring after the 1st vaccination dose (OR = 43; 95% CI = [1.43, >999]; *p* value = 0.030) (Table 2).

## 4. Discussion

The aetiology of GBS remains unknown but is commonly linked to infections or vaccinations [2]. Since the start of COVID-19, there have been cases of GBS being reported after the COVID-19 infection, although a causal link could not be made between the two [44]. With the rollout of vaccination programs, some observational studies [42] demonstrated a potentially small but significant risk of GBS occurring after COVID-19 vaccination [4], with an estimated incidence rate of GBS that is four times the background incidence rate [45]. However, there is still little known about the characteristics, outcomes and the influence of demographic and vaccine factors on clinical outcomes of GBS occurring after COVID-19 vaccination.

Lupica et al. have shown that there was a higher incidence of post-COVID-19 vaccination neurological adverse effects such as Myasthenia Gravis in the female gender and after the first dose [6]. In the same vein, our study highlights for the first time that the female gender is associated with greater clinical severity and worse outcome of GBS linked to COVID-19 vaccination. Previous literature has associated the female gender with an increased risk of side effects following COVID-19 vaccines, citing more vigorous immune responses in females and differences in pain thresholds as possible mechanisms [46]. The differences and effects of sex steroids in modulating immune response between males and females have also been proposed to impact the clinical severity of immune-related pathologies such as GBS [47]. In addition, we found an association linking viral vector vaccines to an earlier onset of GBS presentation and a higher incidence of GBS occurring after the first dose, further linking the more generic systemic side effect profile in viral vector COVID-19 vaccines [46]. These findings will be crucial for future prognostication and pre-emptive management of post-COVID-19 vaccination GBS between different patient groups, but further research would still be useful in exploring the impact of vaccine mechanisms on its side effect characteristics.

From our analysis, most affected patients had classic GBS. Interestingly, there were 12 cases of GBS presenting with facial diplegia, most with associated limb weakness, although there were isolated presentations of facial diplegia [48,49]. Hence it is important for clinicians to have a high index of awareness to identify potential first presentations of a rare but debilitating course of GBS. 

Our study also showed that reported cases of GBS occurring post-COVID-19 vaccination generally displayed positive responses to treatment and post-treatment outcomes, with most patients reporting definite or complete improvement after initiation of treatment and most patients being able to ambulate post-treatment. However, severe outcomes such as respiratory failure and even death have also been observed among the reported cases, emphasizing the potentially debilitating effect of GBS. The long-term post-COVID-19 vaccination neurological side effects associated with GBS are still unclear. A longer trajectory for these neurological side effects could be tracked in the future. Furthermore, subjectivity bias could arise in determining treatment outcomes, which could be improved with future developments of a quantitative scoring system.

Through this study, we have established important links and provided assuring evidence for treatment response and post-treatment outcomes of GBS occurring post-COVID-19 vaccination. While there have been increasing reports of neurological adverse effects occurring post-COVID-19 vaccination, the incidence remains low [5,6]. The COVID-19 vaccination is still largely safe in most patients and should be recommended to prevent other life-threatening complications of COVID-19. While the benefits of COVID-19 vaccination outweigh the risk of severe adverse events such as GBS [50], our findings provide further impetus to study the possible links and mechanisms underpinning the impact of gender and vaccine type on treatment outcomes for GBS occurring post-COVID-19 vaccination. A heightened vigilance for post-vaccine GBS in certain patient groups will be helpful so that timely investigations and treatment can be administered to prevent debilitating outcomes.

## Figures and Tables

**Table 1:** Summary of demographics, vaccine data, onset duration, severity and outcomes of patients with Guillain–Barré Syndrome, *n* = 57.

Variable	Mean (SD)/No. (%)
**Demographics**	
Age (year)	59.5 (14.8)
Age (range)	25, 90
Male gender	31 (54)
**Vaccine Type**	
Viral Vector	
AstraZeneca	34 (60)
Janssen/Johnson & Johnson	5 (9)
mRNA	
Pfizer	15 (26)
Moderna	2 (4)
Inactivated (CoronaVac-SinoVac)	1 (2)
**Vaccine Dose Number**	
First dose	46 (81)
Second dose	8 (14)
Not Reported	3 (6)
**Duration of onset (days)**	
Mean	10.7 (5.2)
Median (IQR)	11 (7, 14)
Early-onset (≤7 days)	17 (30)
Late-onset (≥8 days)	40 (70)
**Severity of Symptoms**	
ICU admission	16 (28)
Respiratory failure	14 (25)
Mechanical ventilation	13 (23)
Death	1 (2)
**Clinical Classification**	
Classic GBS	35 (61)
Paraparetic GBS	9 (16)
Facial diplegia	12 (21)
Cervicobrachial weakness	1 (2)
**Electrodiagnosis**	
Not done	5 (9)
AIDP	37/52 (65)
AMAN	4/52 (8)
AMSAN	8/52 (15)
Equivocal	3/52 (6)
**CSF**	
Not done	5 (9)
Normal	5/52 (10)
Albuminocytological dissociation	47/52 (90)
Protein levels (mean, mg/dL)	211 ± 325
**Treatment**	
IVIG	49 (86)
Plasmapheresis	1 (2)
IVIG and plasmapheresis	2 (4)
Gabapentin	1 (2)
Oral prednisolone	1 (2)
Not done	3 (5)
**Treatment Course**	
Not reported	1 (2)
No improvement	4 (7)
Minimal improvement	16 (28)
Definite improvement	34 (60)
Recovery	2 (4)
**GBS Disability Score Post-treatment**	
NA	6 (11)
0	5/51 (10)
1	14/51 (28)
2	7/51 (14)
3	8/51 (16)
4	11/51 (22)
5	5/51 (10)
6	1/51 (2)

*Abbreviations*: SD = standard deviation; mRNA = messenger Ribonucleic Acid; GBS = Guillain–Barré Syndrome; ICU = intensive care unit; IQR = interquartile range; IVIg = intravenous immune globulin; CSF = cerebrospinal fluid; AIDP = acute inflammatory demyelinating polyneuropathy; AMAN = acute motor axonal neuropathy; AMSAN = acute motor sensory axonal neuropathy; NA = not available.

**Table 2:** Summary of multivariable regression analysis.

Variable	Event vs. Reference *	Vaccine (mRNA vs. Viral Vector) (*n* = 53) ^‡^		Clinical Severity ^†^ (*n* = 54) ^§^		GBS Disability Score (*n* = 48) ^||^	
		OR (95% CI)	*p* Value	OR (95% CI)	*p* Value	β (95% CI)	*p* Value
Sex	Male vs. Female	0.89 (0.19, 4.12)	0.879	0.26 (0.07, 0.91)	0.035	−1.41 (−2.27, −0.55)	0.002
Dose number	2 vs. 1	43 (1.43, >999)	0.030	0.08 (0.003, 1.86)	0.115	−1.11 (−2.31, 0.09)	0.070
Onset	Early vs Late	11.1 (2.35, 52.1)	0.0020	1.41 (0.35, 5.70)	0.629	0.17 (−0.80. 1.14)	0.727

* Inactivated = Corona Vac/Sino Vac; mRNA = Moderna/Pfizer; Viral vector = Janssen Johnson/AstraZeneca; ^†^ Clinical Severity = Respiratory failure or required ventilation or intensive care unit (ICU) admission; ^‡,§^ Multivariable logistic regression analysis is performed to calculate the OR and 95% CI; ^||^ Multivariable linear regression analysis is conducted to calculate the β and 95% CI; ^‡^ There is one patient with received ‘inactivated’ vaccine which was excluded from the analysis; ^‡,§,||^ There are three patients without reported dose number who were excluded from the analysis; ^||^ Six patients without reported GBS disability scores were excluded from the analysis; *Abbreviations*: OR = Odds ratio; CI = Confidence Interval; mRNA = messenger Ribonucleic Acid; GBS = Guillain–Barré syndrome.

## Data Availability

Data is contained within the article or supplementary material.

## Supplementary Materials

The following are available online at https://www.mdpi.com/article/10.3390/brainsci12060711/s1, Table S1. Demographic characteristics and COVID-19 vaccine data of the 57 patients, Table S2. Clinical presentation and severity of the 57 patients, Table S3. Investigation results, treatment, and outcomes of the 57 patients
