# Supplementary material for: Prognosis of Guillain–Barré Syndrome Linked to COVID-19 Vaccination
Source: Brain Sci. 2022 May 30;12(6):711. doi: 10.3390/brainsci12060711 (PMC9220921; doi:10.3390/brainsci12060711)
Supplement: Supplementary file 1 [file brainsci-12-00711-s001.zip › brainsci-1712659-supplementary.pdf]

Supplementary Materials

Annex 1

Database search strategy

PubMed: 88
((Guillain-Barré syndrome) OR (Miller Fisher syndrome) OR (cranial polyneuritis) OR (facial diplegia) OR (Acute sensory ataxia) OR (Bickerstaff encephalitis) OR (acute inflammatory demyelinating polyneuropathy) OR (acute motor axonal neuropathy) OR (acute motor and sensory axonal neuropathy)) AND ((SARS-CoV-2 vaccine) OR (SARS-CoV-2 vaccination) OR (COVID-19 vaccine) OR (COVID-19 vaccination))

Time: 2021-01-01 to 2021-11-01

Web of Science: 69
Step 1: Topic (Guillain-Barré syndrome) OR (Miller Fisher syndrome) OR (cranial polyneuritis) OR (facial diplegia) OR (Acute sensory ataxia) OR (Bickerstaff encephalitis) OR (acute inflammatory demyelinating polyneuropathy) OR (acute motor axonal neuropathy) OR (acute motor and sensory axonal neuropathy)
Step 2: Topic (SARS-CoV-2 vaccine) OR (SARS-CoV-2 vaccination) OR (COVID-19 vaccine) OR (COVID-19 vaccination)

Search: 1 AND 2
Time: 2021-01-01 to 2021-11-01

Embase: 63
Guillain-Barre syndrome AND (SARS-CoV-2 vaccine OR SARS-CoV-2 vaccination OR COVID-19 vaccine OR COVID-19 vaccination)
Time: 2021-01-01 to 2021-11-01

Table S1. Demographic characteristics and COVID-19 vaccine data of the 57 patients.

Author (country)	Age (years)	Gender	Past medical history	Vaccine type	Dose number	Onset duration (days)
Wai et al (Australia)[1]	51	M	NSTEMI, 8 weeks post influenza vaccine	Viral Vector (Astrazeneca)	1	14
	65	F	4 weeks post influenza vaccine	Viral Vector (Astrazeneca)	1	7
	72	M	Idiopathic Neuropathy, 6 weeks post influenza vaccine	Viral Vector (Astrazeneca)	1	21
	66	M	RCC, AF, HLD, 8 weeks post influenza vaccine	Viral Vector (Astrazeneca)	1	21
Grimshaw et al (Mexico)[2]	25	M	Gastrointestinal infection (8 days ago)	mRNA (Pfizer)	1	12
	53	F	Drug allergy, Previous COVID, Norovirus (2 days ago)	mRNA (Pfizer)	1	6
	72	M	Acute Hepatitis (56 days ago)	mRNA (Pfizer)	1	4
	31	M	Nil	mRNA (Pfizer)	1	11
	67	F	Nil	mRNA (Pfizer)	1	4
	81	F	HTN, CKD, 40 days post influenza vaccine	mRNA (Pfizer)	1	3
Hasan et al (UK)[3]	62	F	bronchiectasis, asthma, osteoporosis and migraine	Viral Vector (Astrazeneca)	1	11
Mckean et al (Malta)[4]	48	M	HLD	Viral Vector (Astrazeneca)	1	10
Razok et al (Qatar)[5]	73	M	HTN, RA	mRNA (Pfizer)	2	20
YG Min et al (S. Korea)[6]	58	M	Nil	Viral Vector (Astrazeneca)	1	15
Scendoni et al (Italy)[7]	82	F	AF, HTN, bilateral hip prosthesis	mRNA (Pfizer)	2	14
Suri et al (India)[8]	47	M	DM, HTN, COVID-19 Pneumonia 7 months prior	Viral Vector (Astrazeneca)	1	17

Waheed et al (US)[9]	82	F	Nil	mRNA (Pfizer)	1	14
Tutar et al (Turkey)[10]	76	M	Nil	Inactivated (CoronaVac-SinoVac)	2	14
Maramattom et al (India)[11]	43	F	Nil	Viral Vector (Astrazeneca)	1	10
	67	F	Nil	Viral Vector (Astrazeneca)	1	14
	53	F	Nil	Viral Vector (Astrazeneca)	1	12
	68	F	Nil	Viral Vector (Astrazeneca)	1	14
	70	M	Nil	Viral Vector (Astrazeneca)	1	11
	69	F	Nil	Viral Vector (Astrazeneca)	1	12
	69	F	Nil	Viral Vector (Astrazeneca)	1	13
Introna et al (Italy)[12]	62	M	HTN	Viral Vector (Astrazeneca)	1	10
Rossetti et al (US)[13]	38	M	Anxiety, Depression	Viral Vector (Janssen/Johnson & Johnson)	NA	14
Nasuelli et al (Italy)[14]	59	M	HTN, Hyperuricemia	Viral Vector (Astrazeneca)	NA	10
Hughes et al (US)[15]	65	M	Post Liver Transplant for Cryptogenic Liver Cirrhosis in June 2020, CAD, DM, HLD	mRNA (Pfizer)	1	2
Prasad et al (US)[16]	41	M	Morbid obesity	Viral Vector (Janssen/Johnson & Johnson)	1	21
Allen et al (UK)[17]	54	M	Nil	Viral Vector (Astrazeneca)	1	12
	57	M	Asthma, Osteoarthritis	Viral Vector (Astrazeneca)	1	11
Ogbebor et al (US)[18]	86	F	HTN, RA, DCIS of the breast, osteoporosis	mRNA (Pfizer)	1	1
Bonifacio et al (UK)[19]	43	M	Nil	Viral Vector (Astrazeneca)	1	11

	66	M	Nil	Viral Vector (Astrazeneca)	1	7
	51	M	Nil	Viral Vector (Astrazeneca)	1	21
	71	F	Nil	Viral Vector (Astrazeneca)	1	12
	53	M	Nil	Viral Vector (Astrazeneca)	1	8
Bax et al (Italy)[20]	90	M	HTN, CKD, psoriasis, previous gastric resection and prostatectomy	mRNA (Pfizer)	2	3
	51	F	Nil	Viral Vector (Astrazeneca)	1	10
Matarneh et al (Qatar)[21]	61	M	Nil	mRNA (Moderna)	2	4
Trimboli et al (Italy)[22]	25	F	Nil	mRNA (Pfizer)	2	4
Rao et al (US)[23]	42	F	Nil	mRNA (Pfizer)	2	7
James et al (India)[24]	60	M	COPD	Viral Vector (Astrazeneca)	1	11
	66	M	Nil	Viral Vector (Astrazeneca)	1	12
	54	F	Breast Cancer	Viral Vector (Astrazeneca)	1	13
Kripalani et al (India)[25]	52	F	Nil	Viral Vector (Astrazeneca)	1	9
Patel et al (UK)[26]	37	M	Nil	Viral Vector (Astrazeneca)	1	14
Morehouse et al (US)[27]	49	F	Neurofibromatosis type 1, hypothyroidism, vitamin deficiencies	Viral Vector (Janssen/Johnson & Johnson)	1	5
Kanabar et al (UK)[28]	61	F	Multiple Sclerosis	Viral Vector (Astrazeneca)	1	10
	56	M	Nil	Viral Vector (Astrazeneca)	1	7
Dalwadi et al (US)[29]	86	F	AF, DM, aortic stenosis, ischemic	mRNA (Moderna)	2	2

			stroke (no residue deficits)			
Aomar-Millán et al (Spain)[30]	77	M	Nil	mRNA (Pfizer)	1	3
Jain et al (US)[31]	65	F	HTN, DM, HLD, allergies to ciprofloxacin, codeine, penicillin, and egg whites	Viral Vector (Janssen/Johnson & Johnson)	NA	15
Márquez et al (US)[32]	60	F	Nil	Viral Vector (Janssen/Johnson & Johnson)	1	10
Da Silva et al (Brazil)[33]	62	F	HTN, CCF	Viral Vector (Astrazeneca)	1	18
Shao et al (Taiwan)[34]	41	M	Nil	Viral Vector (Astrazeneca)	1	7

Abbreviations: GBS = Guillain–Barré syndrome; COVID-19 = coronavirus disease 2019; NSTEMI = non-ST-elevation myocardial infarction; RCC = renal cell carcinoma; AF = atrial fibrillation; HTN = hypertension; HLD = hyperlipidaemia; DM = diabetes mellitus; CKD = chronic kidney disease; CAD = coronary artery disease; CCF = congestive cardiac failure; RA = rheumatoid arthritis; DCIS = ductal carcinoma in-situ; COPD = chronic obstructive pulmonary disease.

Table S2. Clinical presentation and severity of the 57 patients.

Author (country)	LL weakness (1 = Yes, 0 = No)	4 Limbs weakness (1 = Yes, 0 = No)	Hypoareflexia (1 = Yes, 0 = No)	Sensory disturbance (1= Yes, 0 = No)	CN involvement (1= Yes, 0 = No)	Dysautonomia (1 = Yes, 0 = No)	Ataxia (1= Yes, 0 = No)	LBP (1= Yes, 0 = No)	Other	GBS clinical classification	Respiratory failure	Ventilation	ICU
Wai et al (Australia)[1]	No	Yes	Yes	Yes	Yes	No	Yes	Yes	No	Classic GBS	Yes	Yes	Yes
	No	Yes	Yes	Yes	Yes	No	No	Yes	No	Classic GBS	Yes	Yes	Yes
	No	Yes	Yes	Yes	No	No	No	No	No	Classic GBS	No	No	No
	Yes	No	Yes	Yes	Yes	No	No	Yes	No	Paraparetic GBS	No	No	No
Grimshaw et al (Mexico)[2]	No	Yes	No	Yes	No	No	No	No	No	Classic GBS	No	No	No
	No	Yes	Yes	No	No	No	No	No	No	Classic GBS	Yes	Yes	Yes
	No	Yes	Yes	No	No	No	No	No	No	Classic GBS	No	No	No
	No	Yes	Yes	No	No	No	No	No	No	Classic GBS	No	No	No
	No	Yes	Yes	No	No	No	No	No	No	Classic GBS	Yes	Yes	Yes
	No	Yes	Yes	No	No	No	No	No	No	Classic GBS	No	No	No
Hasan et al (UK)[3]	No	Yes	Yes	Yes	Yes	No	No	No	No	Classic GBS	Yes	Yes	Yes
Mckean et al (Malta)[4]	Yes	No	Yes	Yes	Yes	No	No	Yes	No	Classic GBS	No	No	No
Razok et al (Qatar)[5]	Yes	No	Yes	No	No	No	No	No	No	Paraparetic GBS	No	No	No
YG Min et al (S. Korea)[6]	No	No	No	Yes	Yes	No	No	No	No	Paraparetic GBS	No	No	No
Scendoni et al (Italy)[7]	No	Yes	Yes	Yes	No	No	No	No	No	Classic GBS	No	No	No
Suri et al (India)[8]	No	Yes	Yes	Yes	Yes	No	No	No	No	Classic GBS	No	No	No

Waheed et al (US)[9]	Yes	No	Yes	Yes	No	No	No	Yes	No	Paraparetic GBS	No	No	No
Tutar et al (Turkey)[10]	No	Yes	Yes	Yes	No	No	No	No	No	Classic GBS	No	No	No
Maramattom et al (India)[11]	No	Yes	Yes	No	Yes	No	No	Yes	No	Classic GBS	Yes	Yes	Yes
	No	Yes	Yes	Yes	Yes	No	No	No	No	Classic GBS	Yes	Yes	Yes
	No	Yes	Yes	Yes	Yes	No	No	No	No	Classic GBS	Yes	Yes	Yes
	No	Yes	Yes	Yes	Yes	No	No	No	No	Classic GBS	Yes	Yes	Yes
	No	Yes	Yes	Yes	Yes	No	No	No	No	Classic GBS	Yes	Yes	Yes
	No	Yes	Yes	Yes	Yes	No	No	No	No	Classic GBS	No	No	No
	No	Yes	Yes	Yes	Yes	No	No	No	No	Classic GBS	Yes	Yes	Yes
Introna et al (Italy)[12]	No	Yes	Yes	Yes	Yes	Yes	Yes	Yes	No	Classic GBS	No	No	Yes
Rossetti et al (US)[13]	No	No	Yes	Yes	Yes	No	No	No	No	Facial diplegia with parasthesia	No	No	No
Nasuelli et al (Italy)[14]	No	No	Yes	Yes	Yes	Yes	Yes	No	No	Facial diplegia with parasthesia	No	No	No
Hughes et al (US)[15]	No	Yes	Yes	Yes	Yes	No	No	No	No	Classic GBS	No	No	No
Prasad et al (US)[16]	No	Yes	Yes	No	Yes	No	Yes	No	No	Classic GBS	No	No	No
Allen et al (UK)[17]	No	No	No	Yes	Yes	No	No	No	No	Facial diplegia with parasthesia	No	No	No
	No	Yes	Yes	Yes	Yes	No	No	Yes	No	Classic GBS	No	No	No
Ogbebor et al (US)[18]	Yes	No	Yes	No	No	No	No	No	No	Paraparetic GBS	No	No	No
Bonifacio et al (UK)[19]	No	No	Yes	Yes	Yes	Yes	Yes	No	No	Facial diplegia with parasthesia	No	No	No
	No	No	Yes	Yes	Yes	No	Yes	Yes	No	Facial diplegia with parasthesia	No	No	No

	No	No	Yes	Yes	Yes	No	Yes	No	No	Facial diplegia with parasthesia	No	No	No
	Yes	No	Yes	No	Yes	No	Yes	Yes	Yes (abdo minal pain)	Facial diplegia with parasthesia	No	No	No
	No	No	Yes	Yes	Yes	No	No	Yes	No	Facial diplegia with parasthesia	No	No	No
Bax et al (Italy)[20]	No	Yes	No	Yes	No	No	Yes	No	No	Classic GBS	No	No	No
	Yes	No	Yes	Yes	Yes	Yes	No	No	No	Paraparetic GBS	No	No	No
Matarneh et al (Qatar)[21]	No	No	No	Yes	No	No	No	No	Yes (distal UL weakn ess)	Cervicobrchial Weakness	No	No	No
Trimboli et al (Italy)[22]	Yes	No	Yes	Yes	No	No	Yes	No	No	Paraparetic GBS	No	No	No
Rao et al (US)[23]	No	Yes	Yes	Yes	No	Yes	No	No	No	Classic GBS	No	No	No
James et al (India)[24]	No	Yes	Yes	Yes	Yes	No	No	No	Yes (pain over torso)	Classic GBS	No	No	No
	No	Yes	Yes	Yes	No	Yes	No	No	Yes	Classic GBS	No	No	No
	No	Yes	Yes	No	No	No	No	No	Yes (cough)	Classic GBS	No	No	No
Kripalani et al (India)[25]	No	Yes	Yes	No	No	No	No	Yes	Yes (fatigu e)	Classic GBS	Yes	Yes	Yes
Patel et al (UK)[26]	No	Yes	Yes	Yes	No	No	Yes	Yes	No	Classic GBS	No	No	Yes
Morehouse et al (US)[27]	No	Yes	Yes	Yes	Yes	Yes	No	Yes	No	Classic GBS	Yes	Yes	Yes

Kanabar et al (UK)[28]	Yes	No	Yes	Yes	Yes	No	No	No	No	Facial diplegia with parasthesia	No	No	No
	No	No	Yes	Yes	Yes	No	No	Yes	No	Facial diplegia with parasthesia	No	No	No
Dalwadi et al (US)[29]	No	Yes	No	No	No	No	No	No	No	Classic GBS	No	No	No
Aomar-Millán et al (Spain)[30]	Yes	No	Yes	Yes	No	No	No	No	Yes (pitting oedema)	Paraparetic GBS	Yes	No	No
Jain et al (US)[31]	No	No	Yes	No	Yes	No	No	No	Yes (headache, neck pain, shoulder pain)	Facial diplegia without parasthesia	No	No	Yes
Márquez et al (US)[32]	Yes	No	Yes	Yes	Yes	No	No	No	Yes (headache, N&V)	Paraparetic GBS	No	No	No
Da Silva et al (Brazil)[33]	No	Yes	Yes	Yes	Yes	Yes	No	No	No	Classic GBS	No	No	No
Shao et al (Taiwan)[34]	No	No	No	Yes	Yes	No	No	No	No	Facial diplegia with parasthesia	No	No	No

Abbreviations: GBS = Guillain–Barré syndrome; CN = cranial nerve; LBP = lower back pain; UL = upper limb; LL = Lower limb.

Table S3. Investigation results, treatment, and outcomes of the 57 patients.

Author (country)	Albuminocytological dissociation (CSF protein, mg/dL)	Electrodiagnosis	Brighton criteria	Antiganglioside Antibody	Treatment	Treatment outcome	GBS disability score (post-treatment outcome)
Wai et al (Australia)[1]	Yes (70)	AIDP	1	NA	IVIg	No improvement	5
	Yes (251)	AIDP	1	Negative	IVIg	Definite improvement	NA
	Yes (55)	AIDP	1	NA	IVIg	Definite improvement	2
	Yes (150)	AIDP	1	NA	IVIg	Definite improvement	NA
Grimshaw et al (Mexico)[2]	Yes (64)	AIDP	1	NA	IVIg	Definite improvement	3
	Yes (15)	AMAN	2	NA	IVIg	Minimal improvement	5
	NA	AMAN	2	NA	IVIg	Definite improvement	4
	NA	AIDP	2	NA	IVIg	Definite improvement	3
	Yes (30)	AMAN	2	NA	IVIg	No improvement	6
	Yes (414)	AIDP	1	NA	IVIg	Definite improvement	4
Hasan et al (UK)[3]	Yes (90)	AIDP	1	NA	IVIg	No improvement	5
Mckean et al (Malta)[4]	Yes (126.4)	AIDP	1	Negative	IVIg	Definite improvement	1
Razok et al (Qatar)[5]	Yes (80)	AIDP	1	NA	IVIg	Definite improvement	0
YG Min et al (S. Korea)[6]	Yes (70)	AIDP	1	Negative	Gabapentin	Minimal improvement	1
Scendoni et al (Italy)[7]	Yes (570)	AMSAN	1	Negative	IVIg	Definite improvement	4
Suri et al (India)[8]	Yes (250)	AIDP	1	Negative	IVIg	Minimal improvement	1

Waheed et al (US)[9]	Yes (88)	NA	2	NA	IVIg	Definite improvement	NA
Tutar et al (Turkey)[10]	NA	AMSAN	2	NA	IVIg	NA	2
Maramattom et al (India)[11]	Yes (85)	AIDP	1	NA	IVIg	Definite improvement	0
	Yes (345)	AMSAN	1	Negative	IVIg	Minimal improvement	4
	Yes (120)	AIDP	1	Negative	IVIg	Minimal improvement	4
	Yes (75)	AIDP	1	Negative	IVIg	Minimal improvement	4
	NA	AIDP	2	NA	IVIg	Minimal improvement	4
	NA	AIDP	2	NA	IVIg	Minimal improvement	4
	Yes (83)	AIDP	2	NA	IVIg	Minimal improvement	5
Introna et al (Italy)[12]	Yes (101)	AMSAN	1	NA	IVIg	Minimal improvement	4
Rossetti et al (US)[13]	Yes (181)	NA	2	NA	IVIg	Minimal improvement	1
Nasuelli et al (Italy)[14]	Yes (140)	AIDP	1	Negative	IVIg	Definite improvement	3
Hughes et al (US)[15]	Yes (107)	AIDP	1	NA	IVIg	Definite improvement	1
Prasad et al (US)[16]	Yes (562)	AIDP	1	NA	IVIg	Definite improvement	3
Allen et al (UK)[17]	Yes (162.6)	AIDP	1	Negative	Oral prednisolone	Definite improvement	1
	Yes (247.1)	Equivocal	2	Negative	IVIg	Definite improvement	1
Ogbebor et al (US)[18]	Yes (162)	NA	2	NA	IVIg	Definite improvement	3
Bonifacio et al (UK)[19]	Yes (281)	AIDP	1	Negative	IVIg	Minimal improvement	1
	Yes (199)	AIDP	1	Negative	IVIg	Definite improvement	2

	Yes (514)	AIDP	1	NA	NA	Definite improvement	2
	Yes (96)	AIDP	1	Negative	NA	Definite improvement	2
	Yes (122)	AIDP	1	Negative	NA	Definite improvement	1
Bax et al (Italy)[20]	Yes (397)	AMSAN	1	NA	IVIg	Definite improvement	1
	Yes (2272)	AIDP	1	Negative	IVIg	Definite improvement	1
Matarneh et al (Qatar)[21]	Yes (50)	AIDP	1	NA	IVIg	Definite improvement	0
Trimboli et al (Italy)[22]	NA	AIDP	2	NA	IVIg	Definite improvement	2
Rao et al (US)[23]	Yes (167)	AIDP	1	NA	IVIg	Definite improvement	1
James et al (India)[24]	Yes (149)	AMSAN	1	NA	IVIg	Minimal improvement	3
	Yes (84)	AIDP	1	NA	IVIg	Definite improvement	3
	NA	AIDP	1	NA	IVIg	Definite improvement	2
Kripalani et al (India)[25]	Yes (91)	AIDP	1	NA	IVIg	No improvement	4
Patel et al (UK)[26]	Yes (177)	Equivocal	2	NA	IVIg	Minimal improvement	NA
Morehouse et al (US)[27]	Yes (58)	AMSAN	1	NA	IVIg	Minimal improvement	5
Kanabar et al (UK)[28]	Yes (164)	AIDP	1	NA	IVIg	Definite improvement	1
	Yes (160)	AIDP	1	NA	IVIg	Recovered	0
Dalwadi et al (US)[29]	Yes (128)	AMAN	1	Negative	Plasmapheresis	Definite improvement	4
Aomar-Millán et al (Spain)[30]	Yes (39)	AMSAN	2	Negative	IVIg, Plasmapheresis	Definite improvement	NA
Jain et al (US)[31]	Yes (302)	NA	2	Positive	IVIg, Plasmapheresis	Recovered	0
Márquez et al (US)[32]	Yes (140)	AIDP	1	Negative	IVIg	Definite improvement	NA

Da Silva et al (Brazil)[33]	Yes (110)	NA	2	NA	IVIg	Minimal improvement	3
Shao et al (Taiwan)[34]	Yes (159.8)	Equivocal	1	NA	IVIg	Definite improvement	1

Abbreviations: GBS = Guillain–Barré syndrome; CSF = cerebrospinal fluid; AIDP = acute inflammatory demyelinating polyneuropathy; AMAN = acute motor axonal neuropathy; AMSAN = acute motor-sensory axonal neuropathy; IVIg = intravenous immune globin; ICU = intensive care unit.

References

1. Alter, M. The epidemiology of Guillain-Barré syndrome. *Ann Neurol*. **1990**, 27, S7-S12.
2. van den Berg, B.; Walgaard, C.; Drenthen, J.; Fokke, C.; Jacobs, B.C.; van Doorn, P.A. Guillain–Barré syndrome: pathogenesis, diagnosis, treatment and prognosis. *Nat Rev Neurol*. **2014**, 10, 469-482.
3. Asbury, A. K.; Cornblath, D. R. Assessment of current diagnostic criteria for Guillain-Barré syndrome. *Ann Neurol*. **1990**, 27.
4. Shao, S.C.; Wang, C.H.; Chang, K.C.; Hung, M.J.; Chen, H.Y.; Liao, S.C. Guillain-Barré Syndrome Associated with COVID-19 Vaccination. *Emerging Infectious Diseases* **2021**, 27, 3175-3178.
5. Hanson, K. E.; Goddard, K.; Lewis, N.; Fireman, B.; Myers, T. R.; Bakshi, N.; Weintraub, E.; Donahue, J. G.; Nelson, J. C.; Xu, S.; Glanz, J. M.; Williams, J. T.; Alpern, J. D.; Klein, N. P. Incidence of Guillain-Barré Syndrome After COVID-19 Vaccination in the Vaccine Safety Datalink. *JAMA Netw Open* **2022**, 5, e228879.
6. Lupica, A.; Di Stefano, V.; Iacono, S.; Pignolo, A.; Quartana, M.; Gagliardo, A.; Fierro, B.; Brighina, F. Impact of COVID-19 in AChR Myasthenia Gravis and the Safety of Vaccines: Data from an Italian Cohort. *Neurol. Int.* **2022**, *14*, 406-416.
7. Khan, F.; Pallant, J.; Ng, L.; Bhasker, A. Factors associated with long-term functional outcomes and psychological sequelae in Guillain–Barre syndrome. *J. Neurol.* **2010**, *257*, 2024-2031.
8. Moher, D.; Liberati, A.; Tetzlaff, J.; Altman, D. Preferred Reporting Items for Systematic Reviews and Meta-Analyses: The PRISMA Statement. *PLoS Med.* **2009**, *6*, e1000097.
9. Wakerley, B.; Uncini, A.; Yuki, N. Guillain–Barré and Miller Fisher syndromes—new diagnostic classification. *Nat. Rev. Neurol.* **2014**, *10*, 537-544.
10. Fokke, C.; van den Berg, B.; Drenthen, J.; Walgaard, C.; van Doorn, P.; Jacobs, B. Diagnosis of Guillain-Barre syndrome and validation of Brighton criteria. *Brain* **2013**, *137*, 33-43.
11. Oo, W.; Giri, P.; de Souza, A. AstraZeneca COVID-19 vaccine and Guillain- Barré Syndrome in Tasmania: A causal link?. *J. Neuroimmunol.* **2021**, *360*, 577719.
12. García-Grimshaw, M.; Michel-Chávez, A.; Vera-Zertuche, J.; Galnares-Olalde, J.; Hernández-Vanegas, L.; Figueroa-Cucurachi, M.; Paredes-Ceballos, O.; Reyes-Terán, G.; Carbajal-Sandoval, G.; Ceballos-Liceaga, S.; Arauz, A.; Valdés-Ferrer, S. Guillain-Barré syndrome is infrequent among recipients of the BNT162b2 mRNA COVID-19 vaccine. *Clin. Immunol.* **2021**, *230*, 108818.
13. Hasan, T.; Khan, M.; Khan, F.; Hamza, G. Case of Guillain-Barré syndrome following COVID-19 vaccine. *BMJ Case Rep.* **2021**, *14*, e243629.
14. McKean, N.; Chircop, C. Guillain-Barré syndrome after COVID-19 vaccination. *BMJ Case Rep.* **2021**, *14*, e244125.
15. Razok, A.; Shams, A.; Almeer, A.; Zahid, M. Post-COVID-19 vaccine Guillain-Barré syndrome; first reported case from Qatar. *Ann. Med. Surg.* **2021**, *67*, 102540.
16. Min, Y.; Ju, W.; Ha, Y.; Ban, J.; Lee, S.; Sung, J.; Shin, J. Sensory Guillain-Barre syndrome following the ChAdOx1 nCov-19 vaccine: Report of two cases and review of literature. *J. Neuroimmunol.* **2021**, *359*, 577691.
17. Scendoni, R.; Petrelli, C.; Scaloni, G.; Logullo, F. Electromyoneurography and laboratory findings in a case of Guillain-Barré syndrome after second dose of Pfizer COVID-19 vaccine. *Hum. Vaccin. Immunother.* **2021**, *17*, 4093-4096.

18. Suri, V.; Pandey, S.; Singh, J.; Jena, A. Acute-onset chronic inflammatory demyelinating polyneuropathy after COVID-19 infection and subsequent ChAdOx1 nCoV-19 vaccination. *BMJ Case Rep.* **2021**, *14*, e245816.
19. Waheed, S.; Bayas, A.; Hindi, F.; Rizvi, Z.; Espinosa, P. Neurological Complications of COVID-19: Guillain-Barre Syndrome Following Pfizer COVID-19 Vaccine. *Cureus* **2021**, 13.
20. Tutar, N.; Eyigürbüz, T.; Yildirim, Z.; Kale, N. A variant of Guillain-Barre syndrome after SARS-CoV-2 vaccination: AMSAN. *Ideggyógy. Sz.* **2021**, *74*, 286-288.
21. Maramattom, B.; Krishnan, P.; Paul, R.; Vishnu, S.C. Guillain-Barré Syndrome following ChAdOx1-S / nCoV -19 Vaccine. *Ann. Neurol.* **2021**, 90, 312-314.
22. Introna, A.; Caputo, F.; Santoro, C.; Guerra, T.; Ucci, M.; Mezzapesa, D.; Trojano, M. Guillain-Barré syndrome after AstraZeneca COVID-19-vaccination: A causal or casual association?. *Clin. Neurol. Neurosurg.* **2021**, *208*, 106887.
23. Rossetti, A.; Gheihman, G.; O'Hare, M.; Kosowsky, J. Guillain-Barré Syndrome Presenting as Facial Diplegia after COVID-19 Vaccination: A Case Report. *J. Emerg. Med* **2021**, *61*, e141-e145.
24. Nasuelli, N.; De Marchi, F.; Cecchin, M.; De Paoli, I.; Onorato, S.; Pettinaroli, R.; Savoini, G.; Godi, L. A case of acute demyelinating polyradiculoneuropathy with bilateral facial palsy after ChAdOx1 nCoV-19 vaccine. *Neurol. Sci.* **2021**, *42*, 4747-4749.
25. Hughes, D.; Brunn, J.; Jacobs, J.; Todd, P.; Askari, F.; Fontana, R. Guillain-Barré Syndrome After COVID-19 mRNA Vaccination in a Liver Transplantation Recipient With Favorable Treatment Response. *Liver Transplant.* **2021**, *28*, 134-137.
26. Prasad, A.; Hurlburt, G.; Podury, S.; Tandon, M.; Kingree, S.; Sriwastava, S. A Novel Case of Bifacial Diplegia Variant of Guillain-Barré Syndrome Following Janssen COVID-19 Vaccination. *Neurol. Int.* **2021**, *13*, 404-409.
27. Allen, C.; Ramsamy, S.; Tarr, A.; Tighe, P.; Irving, P.; Tanasescu, R.; Evans, J. Guillain–Barré Syndrome Variant Occurring after SARS-CoV-2 Vaccination. *Ann. Neurol.* **2021**, *90*, 315-318.
28. Ogbebor, O.; Seth, H.; Min, Z.; Bhanot, N. Guillain-Barré syndrome following the first dose of SARS-CoV-2 vaccine: A temporal occurrence, not a causal association. *Idcases.* **2021**, *24*, e01143.
29. Bonifacio, GB.; Patel, D.; Cook, S.; et al. Bilateral facial weakness with paraesthesia variant of Guillain-Barré syndrome following Vaxzevria COVID-19 vaccine. *J. Neurol. Neurosurg.* **2021**, *93*, 341-342.
30. Bax, F.; Gigli, GL.; Belgrado, E.; Brunelli, L.; Valente, M. Guillain–Barré syndrome following Covid-19 immunization: a report of two cases. *Acta Neurol. Belg.* **2021**, 1-3.
31. Matarneh, A.; Al-battah, A.; Farooqui, K.; Ghamoodi, M.; Alhatou, M. COVID-19 vaccine causing Guillain-Barre syndrome, a rare potential side effect. *Clin. Case Rep.* **2021**, *9*, e04756.
32. Trimboli, M.; Zoleo, P.; Arabia, G.; Gambardella, A. Guillain-Barré syndrome following BNT162b2 COVID-19 vaccine. *Neurol. Sci.* **2021**, *42*, 4401-4402.
33. Rao, S.; Khurana, S.; Murthy, G.; Dawson, E.; Jazebi, N.; Haas, C. A case of Guillain–Barre syndrome following Pfizer COVID-19 vaccine. *J. Community Hosp. Intern. Med. Perspect.* **2021**, *11*, 597-600.
34. James, J.; Jose, J.; Gafoor, V.; Smita, B.; Balaram, N. Guillain-Barré syndrome following ChAdOx1 nCoV-19 COVID-19 vaccination: A case series. *Neurol. Clin. Neurosci.* **2021**, *9*, 402-405.

35. Kripalani, Y.; Lakkappan, V.; Parulekar, L.; Shaikh, A.; Singh, R.; Vyas, P. A Rare Case of Guillain-Barré Syndrome following COVID-19 Vaccination. *Eur. J. Case Rep. Intern. Med.* **2021**, *8*.
36. Patel, S.; Khurram, R.; Lakhani, A.; Quirk, B. Guillain-Barre syndrome following the first dose of the chimpanzee adenovirus-vectored COVID-19 vaccine, ChAdOx1. *BMJ Case Rep.* **2021**, *14*, e242956.
37. Morehouse, Z.; Paulus, A.; Jasti, S.; Bing, X. A Rare Variant of Guillain-Barre Syndrome Following Ad26.COV2.S Vaccination. *Cureus.* **2021**, *13*, e18153.
38. Kanabar, G.; Wilkinson, P. Guillain-Barré syndrome presenting with facial diplegia following COVID-19 vaccination in two patients. *BMJ Case Rep.* **2021**, *14*, e244527.
39. Dalwadi, V.; Hancock, D.; Ballout, A.; Geraci, A. Axonal-Variant Guillian-Barre Syndrome Temporally Associated With mRNA-Based Moderna SARS-CoV-2 Vaccine. *Cureus.* **2021**, *13*, e18291.
40. Aomar-Millán, I.; Martínez de Victoria-Carazo, J.; Peregrina-Rivas, J.; Villegas-Rodríguez, I. COVID-19, Guillain-Barré syndrome, and the vaccine. A dangerous combination. *Rev Clin Esp.* **2021**, *221*, 555-557.
41. Jain, E.; Pandav, K.; Regmi, P.; Michel, G.; Altshuler, I. Facial Diplegia: A Rare, Atypical Variant of Guillain-Barré Syndrome and Ad26.COV2.S Vaccine. *Cureus.* **2021**, *13*, e16612.
42. Márquez Loza, A.; Holroyd, K.; Johnson, S.; Pilgrim, D.; Amato, A. Guillain-Barré Syndrome in the Placebo and Active Arms of a COVID-19 Vaccine Clinical Trial. *Neurol.* **2021**, *96*, 1052-1054.
43. Silva, G.; Silva, C.; Oliveira, R; et al. Guillain–Barré syndrome after coronavirus disease 2019 vaccine: A temporal association. *Clin. Exp. Neuroimmunol.* **2021**.
44. Shao, S.; Wang, C.; Chang, K.; Hung, M.; Chen, H.; Liao, S. Guillain-Barré Syndrome Associated with COVID-19 Vaccination. *Emerging Infect. Dis.* **2021**, *27*, 3175-3178.
45. Lunn, MP.; Cornblath. DR.; Jacobs, BC.; et al. COVID-19 vaccine and Guillain-Barré syndrome: let's not leap to associations. *Brain*. **2021**, *144*, 357-360.
46. Woo, E.; Mba-Jonas, A.; Dimova, R.; Alimchandani, M.; Zinderman, C.; Nair, N. Association of Receipt of the Ad26.COV2.S COVID-19 Vaccine With Presumptive Guillain-Barré Syndrome, February-July 2021. *JAMA.* **2021**, *326*, 1606.
47. Klugar, M,.; Riad, A.; Mekhemar, M.; et al. Side effects of mRNA-based and viral vector-based COVID-19 vaccines among German healthcare workers. *Biology.* **2021**, *10*, 752.
48. Klein, S.; Jedlicka, A.; Pekosz, A. The Xs and Y of immune responses to viral vaccines. *Lancet Infect. Dis.* **2010**, *10*, 338-349.
49. Iftikhar, H.; Noor, S. U.; Masood, M.; et al. Bell’s Palsy After 24 Hours of mRNA-1273 SARS-CoV-2 Vaccine. *Cureus*. **2021**, *13*, e15935.
50. Wan, EYF.; Chui, CSL.; Lai, FTT.; et al. Bell's palsy following vaccination with mRNA (BNT162b2) and inactivated (CoronaVac) SARS-CoV-2 vaccines: a case series and nested case-control study. *Lancet Infect. Dis*. **2022**, *22*, 64-72.
51. Goodman, JL.; Grabenstein, JD.; Braun, MM. Answering key questions about COVID-19 vaccines. *JAMA*. **2020**, *324*, 2027–2028.
